# Multi-Similarities Bilinear Matrix Factorization-Based Method for Predicting Human Microbe–Disease Associations

**DOI:** 10.3389/fgene.2021.754425

**Published:** 2021-10-14

**Authors:** Xiaoyu Yang, Linai Kuang, Zhiping Chen, Lei Wang

**Affiliations:** ^1^ Key Laboratory of Hunan Province for Internet of Things and Information Security, Xiangtan University, Xiangtan, China; ^2^ College of Computer Engineering and Applied Mathematics, Changsha University, Changsha, China

**Keywords:** microbe, disease, association prediction, multi-similarities, matrix factorization

## Abstract

Accumulating studies have shown that microbes are closely related to human diseases. In this paper, a novel method called MSBMFHMDA was designed to predict potential microbe–disease associations by adopting multi-similarities bilinear matrix factorization. In MSBMFHMDA, a microbe multiple similarities matrix was constructed first based on the Gaussian interaction profile kernel similarity and cosine similarity for microbes. Then, we use the Gaussian interaction profile kernel similarity, cosine similarity, and symptom similarity for diseases to compose the disease multiple similarities matrix. Finally, we integrate these two similarity matrices and the microbe-disease association matrix into our model to predict potential associations. The results indicate that our method can achieve reliable AUCs of 0.9186 and 0.9043 ± 0.0048 in the framework of leave-one-out cross validation (LOOCV) and fivefold cross validation, respectively. What is more, experimental results indicated that there are 10, 10, and 8 out of the top 10 related microbes for asthma, inflammatory bowel disease, and type 2 diabetes mellitus, respectively, which were confirmed by experiments and literatures. Therefore, our model has favorable performance in predicting potential microbe–disease associations.

## Introduction

Microorganisms are the general names of all tiny organisms that individuals cannot observe with the naked eye, but are closely related to humans. Microorganisms include bacteria, viruses, fungi, and a large group of small protozoa, microalgae (The Human Microbiome Project Consortium, 2012). We all know that microbes can cause diseases and make food, cloth, and leather moldy and decay, but it also has a beneficial side. For instance, probiotics in the gut are beneficial to ferment undigested carbohydrates in order to produce nutrition needed for the human body. One of the most important effects of microbes on human beings is to lead to the spread of infectious diseases. Viruses are the cause of 50% of human diseases, therefore, microbes can greatly influence human health. For example, *Mycobacterium tuberculosis* and *Bacillus anthracis* can cause tuberculosis and anthrax, respectively (Hawn et al., 2014; Hendricks et al., 2014). Therefore, identifying disease-related microbes is one of the important tasks in the study of complex disease pathology. One of the useful values of biological research is its application in the field of medicine for the benefit of human health. Identification and prediction of human microbe–disease associations are important for disease prevention, diagnosis, treatment, and prognosis. Nevertheless, the traditional test methods are time consuming and costly. As the result, it is crucial to predict microbe–disease associations by computational methods.

Due to the rapid development of artificial intelligence (AI) and machine learning technology (Huang, 1996; Huang, 1999; Huang and Du, 2008), many computational methods are widely applied in predicting the potential correlation among biological entities [such as miRNA-disease (Chen and Yan, 2015; You et al., 2017; Chen et al., 2018a; Chen et al., 2018b), lncRNA-disease (Chen and Yan, 2013; Chen et al., 2016b; Yu et al., 2018; Chen et al., 2019; Xuan et al., 2019), and drug–target interaction prediction (Chen et al., 2012)]. Meanwhile, many computational methods have been proposed to predict microbe–disease associations. According to the introduction of this paper (Wen et al., 2021), the existing methods can be divided into five categories, namely, path-based methods, random walk methods, bipartite local models, matrix factorization methods, and other methods. The path-based method mainly calculates the relationship between microbe and disease by two indexes, one is walk length, the other is the number of paths reached. KATZHMDA (Chen et al., 2016a), based on path-based method, is the first calculation method by computing the number of walks of connections between microbe and disease nodes in the microbe–disease association network. Random walk methods first construct a transition probability network by microbe and disease nodes; a potential association is then searched by measuring the path probability of the walker from the start node to the end node in the network. BiRWHMDA (Zou et al., 2017), BiRWMP (Shen et al., 2018), and NBLPIHMDA (Wang et al., 2019) using random walk achieves satisfying performance. Bipartite local models calculate the prediction scores of microbes and diseases, respectively, and then the two scores are combined as the final prediction score. Matrix factorization methods decompose an interaction matrix into two low dimensional matrices representing disease features and microbe features. Finally, the product of the two feature matrices is taken as the final prediction matrix. CMFHMDA (Shen et al., 2017) is the first calculation model based on matrix factorization by integrating known microbe–disease association and Gaussian interaction profile kernel similarity for microbes and diseases. MDLPHMDA (Qu et al., 2019) puts forward the matrix decomposition and label propagation to predict microbe–disease association. NMFMDA (Liu et al., 2018) predicts potential associations by graph-regularized non-negative matrix factorization. Other methods mainly include ensemble learning and matrix completion, such as ABHMDA (Peng et al., 2018), BMCMDA (Shi et al., 2018), and MCHMDA (Yan et al., 2021). What is more, the methods based on matrix decomposition were developed to predict the relationship between other biological entities (Wang and Gao, 2015; Qiu et al., 2021a; Qiu et al., 2021b), for example, Qiu et al. (2021a) proposed a novel model based on weighted data fusion with sparse matrix tri-factorization to predict associations between RNA-binding proteins and alternative splicing, namely, WDFSMF. WDFSMF simultaneously decomposes heterogeneous data source matrices into low-rank matrices to mine potential associations.

**FIGURE 1:**
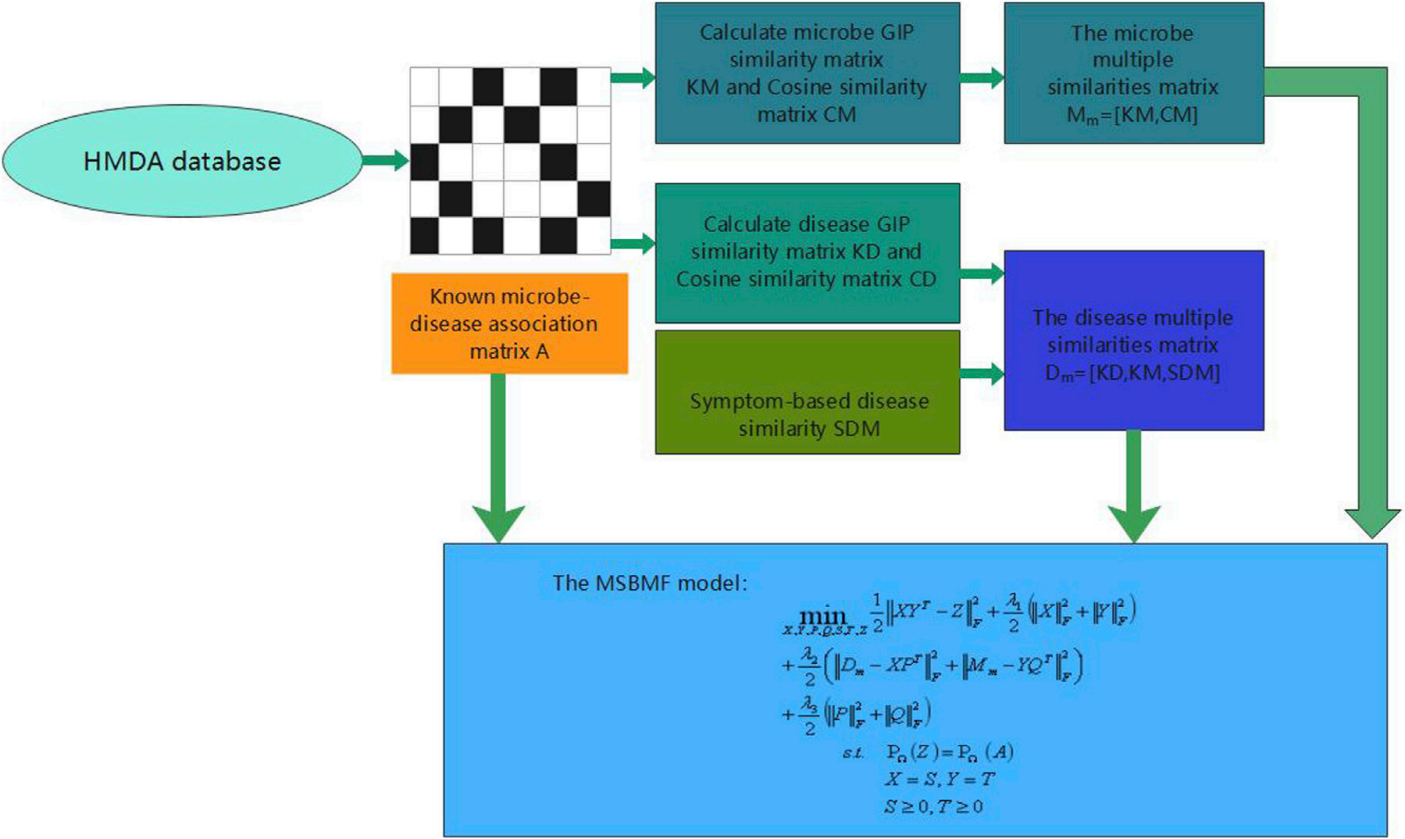
The overall workflow of MSBMFHMDA.

However, some of the above prediction models of microbe–disease have their own limitations. Owing to the lack of measurements for microbe and disease similarity, some models, which are only based on the Gaussian interaction profile kernel similarity of microbes and diseases, cannot be used to predict diseases that are not associated with microbes. In this study, considering the above limitations and inspired by the good performance of multi-similarities bilinear matrix factorization method to predict drug-associated indications (Yang et al., 2021), we proposed a new microbe–disease association prediction model called MSBMFHMDA. The overall workflow of our method is illustrated in Figure 1. First, we calculated the Gaussian interaction profile kernel similarity and cosine similarity for diseases and microbes based on the dataset of known microbe–disease associations. Then, two concatenated microbe and disease similarity matrices are constructed based on the Gaussian interaction profile kernel similarity for diseases and microbes, disease symptom similarity, cosine similarity for diseases, and microbes. Notably, we concatenate these similarity matrices of microbe and disease instead of fusing multiple similarities into a single similarity matrix. Finally, we integrate these two concatenated similarity matrices and the microbe–disease association matrix into our MSBMF model to infer potential microbe–disease associations. The framework of LOOCV and fivefold cross validation were implemented to estimate the prediction performances of MSBMFHMDA. The results suggested that our method could achieve reliable AUCs of 0.9186 and 0.9043 ± 0.0048 in LOOCV and fivefold cross validation, respectively, which is much better than state-of-the-art methods. Moreover, we further implemented the case studies of asthma, IBD, and T2D on MSBMFHMDA, and the reliability of our model is further verified.

## Materials and Methods

### Datasets

The Human Microbe–Disease Association Database (HMDAD) (Ma et al., 2017) is the first human microbe–disease association database established by Ma et al. through a lot of biological experiments. The database includes 483 experimentally tested and verified associations between 292 microbes and 39 diseases. We downloaded the data from HMDAD (http://www.cuilab.cn/hmdad), then removed redundant associations. Thus, 450 microbe–disease associations including 39 diseases and 292 microbes were obtained from 61 publications. As a result, a 39 × 292 dimensional adjacency matrix A is constructed. In addition, in the adjacency matrix A, the value of A[i][j] is set to 1 if microbe m[j] is related to disease d[i] , otherwise, A[i][j] is set to 0.

### Similarity Measures of Microbe

#### Gaussian Interaction Profile Kernel Similarity of Microbes: *KM*

Gaussian kernel function is a common kernel function. Its essence is to measure the similarity between samples (van Laarhoven et al., 2011). It is based on the assumption that two similar diseases and the same microbe will exhibit the same interaction and non-interaction relationship. Therefore, in the known microbe–disease association network, we adopt the Gaussian interaction profile kernel similarity to compute microbe similarity according to the following Eq. 1:

$$\text{KM}\left(m\left(i\right),m\left(j\right)\right)=\exp\left(-\gamma_{m}IP(m\left(i\right)-IP(m\left(j\right))\|2\right)$$ (1)

where m[i] and m[j] represent the ith and jth microbes, respectively, in the matrix *A*, and its interaction profiles IP(m(i)) and IP(m(j)) represent the ith and jth column, respectively. Based on this information, we can calculate the similarity between the two microbe vectors by calculating the *L2* norm. Additionally, the parameter $\gamma_{\text{m}}$ can be calculated as follows:

$$\gamma_{\text{m}}=\frac{\gamma_{m}^{\prime }}{\left(\frac{1}{n_{m}}{\sum_{k=1}^{n_{m}}\left\|IP\left(m\left(k\right)\right)\right\|}^{2}\right)}$$ (2)

where $\gamma_{\text{m}}$ is a parameter used to control the bandwidth of the Gaussian kernel function; it is the result of normalization by bandwidth parameter $\gamma_{m}^{\prime }$ , and according to the previous experiment (van Laarhoven et al., 2011), $\gamma_{m}^{\prime }$ will be set to 1. $n_{m}$ is the total number of microbes collected from the HMDAD, so, $n_{m}$ is equal to 292.

#### Cosine Similarity of Microbes: *CM*

Microbe cosine similarity is calculated based on assumptions that if the microbes are similar to each other (Xie et al., 2019). In other words, in the microbe–disease association matrix, A(i,∶) and A(j,∶) should be similar to each other. Therefore, the cosine similarity between microbe m(i) and microbe m(j) can be calculated as follows:

$$CM\left(m\left(i\right),m\left(j\right)\right)=\frac{A\left(i,:\right)\cdot A\left(j,:\right)}{\left\|A\left(i,:\right)\right\|\times \left\|A\left(j,:\right)\right\|}$$ (3)

where A(i,∶) represents the ith row of adjacency matrix *A*; the result is then projected into [0, 1] by the min–max normalization.

### Similarity Measures of Disease

#### Gaussian Interaction Profile Kernel Similarity of Diseases: *KD*

In a similar way, the Gaussian interaction profile kernel similarity between disease d(i) and disease d(j) can be defined as follows:

$$\text{KD}\left(\text{d}\left(i\right),\text{d}\left(j\right)\right)=\exp\left(-\gamma_{\text{d}}{IP\left(\text{d}\left(i\right)-IP(\text{d}\left(j\right)\right)\|}^{2}\right)$$ (4)

$$\gamma_{\text{d}}=\frac{\gamma_{\text{d}}^{\prime }}{\left(\frac{1}{n_{\text{d}}}{\sum_{k=1}^{n_{\text{d}}}\left\|IP\left(\text{d}\left(k\right)\right)\right\|}^{2}\right)}$$ (5)

$\gamma_{d}^{\prime }$ will be also set to 1; ${\text{n}}_{\text{d}}$ is equal to 39.

#### Cosine Similarity of Diseases: *CD*

The cosine similarity between disease d(i) and disease d(j) is given as follows:

$$C\text{D}\left(\text{d}\left(i\right),\text{d}\left(j\right)\right)=\frac{A\left(:,\text{i}\right)\cdot A\left(:,\text{j}\right)}{\left\|A\left(:,\text{i}\right)\right\|\times \left\|A\left(:,\text{j}\right)\right\|}$$ (6)

where A(∶,i) represents the ith column of adjacency matrix *A*; the result is then projected into [0, 1] by the min–max normalization.

### Symptom-Based Disease Similarity: *SDM*

The abnormal subjective feeling or some objective pathological changes of patients caused by a series of abnormal changes in function, metabolism, and morphological structure in the process of disease are called symptoms. Some diseases, especially in the early stage of some diseases, may not be accompanied by symptoms and signs. The human symptoms–disease network (HSDN) has been constructed by Zhou et al. from PubMed (Wheeler et al., 2007; Zhou et al., 2014). Moreover, they used term frequency inverse document frequency (TF-IDF) (Salton et al., 1975) to measure the symptom-based disease similarity based on the co-occurrence frequency between a disease and a symptom. Based on these data, Chen et al. (2016a) extracted those symptom-based similarities of common diseases from HMDAD. Hence, symptom similarity SDM can be constructed.

### MSBMF Model

As the microbe–disease association matrix is low rank, in other words, it is very sparse, microbe-disease association matrix can be split into two low-dimensional feature matrices, i.e., disease feature X and microbe Y. Then, Tikhonov regularization terms are used to avoid over-fitting. The elementary matrix factorization model is formulated as follows:

$$\underset{X,Y}{\min}\frac{1}{2}{\left\|P_{\Omega }\left(XY^{T}-A\right)\right\|}_{F}^{2}+\frac{\lambda_{1}}{2}\left({\left\|X\right\|}_{F}^{2}+{\left\|Y\right\|}_{F}^{2}\right)$$ (7)

where $\;{•}_{F}$ denotes the Frobenius norm, $A_{F\;}=\sqrt{tr\left(A^{T}A\right)}=\sqrt{\overset{m}{\underset{i=1}{\sum }}\overset{n}{\underset{j=1}{\sum }}a_{ij}^{2}}$ ,  tr(A) is the trace of matrix A, $A_{F}^{2}=tr\left(A^{T}A\right)=\overset{m}{\underset{i=1}{\sum }}\overset{n}{\underset{j=1}{\sum }}a_{ij}^{2}$ , $\lambda_{1}$ is the harmonic parameter to counterpoise the error term and the regularization terms, Ω is an index set of known association in matrix *A*, and ${Ρ}_{\Omega }$ is defined as:

$${\left(P_{\Omega }\left(I\right)\right)}_{ij}=\{\begin{array}{cc} I_{ij}, & \left(i,j\right)\in \Omega \\ 0, & \left(i,j\right)\notin \Omega \end{array}$$ (8)

However, Eq. 7 does not involve prior information about diseases and microbes. Given a disease similarity matrix *D* and a microbe similarity matrix *M*, as *X*,*Y* can be considered as matrices containing disease and microbe potential characteristic vectors, respectively, $XX^{T}$ and $YY^{T}$ are expected to match *D* and *M*, respectively (Zheng et al., 2013; Cui et al., 2019). Therefore, Eq. 7 is extended to:

$$\underset{X,Y}{\min}\frac{1}{2}{\left\|P_{\Omega }\left(XY^{T}-A\right)\right\|}_{F}^{2}+\frac{\lambda_{1}}{2}\left({\left\|X\right\|}_{F}^{2}+{\left\|Y\right\|}_{F}^{2}\right)+\frac{\lambda_{2}}{2}\left({\left\|D-XX^{T}\right\|}_{F}^{2}+{\left\|M-YY^{T}\right\|}_{F}^{2}\right)$$ (9)

In order to incorporate multiple similarity measures, an MSBMF model can be proposed for predicting microbe–disease associations, which is formulated as follows:

$$\begin{array}{l} \underset{X,Y,P,Q,Z}{\min}\frac{1}{2}{\left\|XY^{T}-Z\right\|}_{F}^{2}+\frac{\lambda_{1}}{2}\left({\left\|X\right\|}_{F}^{2}+{\left\|Y\right\|}_{F}^{2}\right)\\ \;\;\;\;\;\;\;\;\;\;+\frac{\lambda_{2}}{2}\left({\left\|D_{m}-XP^{T}\right\|}_{F}^{2}+{\left\|M_{m}-YQ^{T}\right\|}_{F}^{2}\right)\\ \;\;\;\;\;\;\;\;\;\;+\frac{\lambda_{3}}{2}\left({\left\|P\right\|}_{F}^{2}+{\left\|Q\right\|}_{F}^{2}\right)\\ \;\;\;\;\;\;\;\;\;\;s.t.\;\;\;P_{\Omega }\left(A\right)=P_{\Omega }\left(Z\right)\\ \;\;\;\;\;\;\;\;\;\;\;\;\;\;\;\;X\geq 0,Y\geq 0 \end{array}$$ (10)

$D_{m}$ and ${\text{M}}_{m}$ are multi-similarities matrices of diseases and microbes, respectively, and $\lambda_{1}$ , $\lambda_{2}$ , $\lambda_{3}$ are balancing parameters. Obviously, $D_{m}=\left[KD,CD,SDM\right]$ and ${\text{M}}_{\text{m}}=\left[KM,CM\right]$ , where *P* and *Q* are matrices including latent features representing disease similarity and microbe similarity, respectively. *Z* is an auxiliary matrix that helps to optimize. Furthermore, by introducing two splitting matrices *S* and *T*, Eq. 10 is transformed into:

$$\begin{array}{l} \underset{X,Y,P,Q,S,T,Z}{\min}\frac{1}{2}{\left\|XY^{T}-Z\right\|}_{F}^{2}+\frac{\lambda_{1}}{2}\left({\left\|X\right\|}_{F}^{2}+{\left\|Y\right\|}_{F}^{2}\right)\\ \;\;\;\;\;\;\;\;\;\;+\frac{\lambda_{2}}{2}\left({\left\|D_{m}-XP^{T}\right\|}_{F}^{2}+{\left\|M_{m}-YQ^{T}\right\|}_{F}^{2}\right)\\ \;\;\;\;\;\;\;\;\;\;+\frac{\lambda_{3}}{2}\left({\left\|P\right\|}_{F}^{2}+{\left\|Q\right\|}_{F}^{2}\right)\\ \;\;\;\;\;\;\;\;\;\;s.t.\;\;\;P_{\Omega }\left(A\right)=P_{\Omega }\left(Z\right)\\ \;\;\;\;\;\;\;\;\;\;\;\;\;\;\;\;\;S=X,T=Y\\ \;\;\;\;\;\;\;\;\;\;\;\;\;\;\;\;\;S\geq 0,T\geq 0 \end{array}$$ (11)

Then, we use the alternating direction method of multipliers (ADMM) framework to solve Eq. 10. The augmented Lagrangian function is given by:

$$\begin{array}{l} \ell \left(\text{X},Y,P,Q,S,T,Z\right)=\frac{1}{2}{\left\|XY^{T}-Z\right\|}_{F}^{2}+\frac{\lambda_{1}}{2}\left({\left\|X\right\|}_{F}^{2}+{\left\|Y\right\|}_{F}^{2}\right)\\ +\frac{\lambda_{2}}{2}\left({\left\|D_{m}-XP^{T}\right\|}_{F}^{2}+{\left\|M_{m}-YQ^{T}\right\|}_{F}^{2}\right)+\frac{\lambda_{3}}{2}\left({\left\|P\right\|}_{F}^{2}+{\left\|Q\right\|}_{F}^{2}\right)\;\;\;\;\;\;\;\;\\ +\langle \Phi ,X-S\rangle +\langle \Psi ,Y-T\rangle +\frac{\mu }{2}\left({\left\|X-S\right\|}_{F}^{2}+{\left\|Y-T\right\|}_{F}^{2}\right) \end{array}$$ (12)

where Φ and Ψ are the Lagrange multipliers, and μ is the penalty parameter. After k iteration, $X_{k+1},Y_{k+1},P_{k+1},Q_{k+1,}S_{k+1},T_{k+1}\;andZ_{k+1}$ will be computed. We adopt a scheme with gradually increasing learning rate to achieve fast convergence (Shang et al., 2018). After executing the MSBMF algorithm, a non-negative matrix M* is a predicted scores matrix. The scheme of MSBMF model is illustrated in **Algorithm 1**.

Algorithm 1 MSBMF algorithm.

Input: the microbe–disease association matrix M, the multiply similarities of disease matrices *D*_*m*_, the multiply similarities of microbe matrices M_m_, subspace dimensionality r, parameters $\lambda_{1}$ , $\lambda_{2}$ and $\lambda_{3}$ .
Output: predicted association matrix M*.
Step1: calculate microbe GIP similarity and cosine similarity;
Step2: calculate disease GIP similarity, cosine similarity, and symptom-based similarity;
Step 3: initializing randomly four non-negative matrices X_0_, Y_0_, P_0_, Q_0_; S_0_ = X_0_,T_0_ = Y_0_, Z_0_ = M, $\Phi_{0}$ = 0, $\Psi_{0}$ = 0, $\mu_{0}$ , $\mu_{\text{max}}$ , and rate changing factor ρ > 1;
Step4: repeat compute X_k+1_, Y_k+1_, P_k+1_, Q_k+1_, S_k+1_, T_k+1_, and Z_k+1,_ and update the multipliers by: $\Phi_{\text{k}+1}\leftarrow \Phi_{\text{k}}+\mu_{\text{k}}\left({\text{X}}_{\text{k}+1}{-\text{S}}_{\text{k}+1}\right)$ ; $\Psi_{\text{k}+1}\leftarrow \Psi_{\text{k}}+\mu_{\text{k}}\left({\text{Y}}_{\text{k}+1}{-\text{T}}_{\text{k}+1}\right)$ ; update $\mu_{\text{k}+1}$ by $\mu_{\text{k}+1}\leftarrow \text{min}\left(\rho \mu_{\text{k}},\mu_{\text{max}}\right)$ ; k←k+1 ; until convergence;
Step5: obtain the predicted association matrix M*.
Step6: Return M*.

## Results

### Performance Evaluation

The problem of microbe–disease associations prediction can be seen as a classification or regression problem, usually using cross-validation to evaluate the generalization capabilities of the new sample. In order to evaluate performance of our model, we carry out two kinds of computational experiments, including LOOCV and fivefold cross validation. In LOOCV, each confirmed microbe–disease association was chosen as a test sample in turn, and the rest of the associations were used to train. After executing MSBMFHMDA, the score of the test example would be ranked with the scores of candidate samples that were made up of all unconfirmed microbe–disease pairs. In fivefold cross validation, we first divided the known microbe–disease associations into five equal parts and later made each part as a test sample in turn and the remaining four parts of associations as training samples. Similarly, the score of each test sample would be ranked with the scores of candidate samples that were made up of all unconfirmed microbe–disease pairs. As the sample divisions may cause bias, we repeated the fivefold cross-validation 100 times to get an average value as the final result. As the predicted score that obtained a higher rank than the given threshold, our model is considered to make a successful prediction. Then according to diverse thresholds, we plotted the receiver operating characteristics (ROC) curve by computing the ratio of true positive rate (TPR, sensitivity) to false positive rate (FPR, 1-specificity). The AUC can be used to evaluate its predictive performance, where the AUC value of 1 represents perfect prediction ability, and the AUC value of 0.5 indicates random prediction performance (Chen et al., 2016a).

### Effects of the Parameters

In our algorithm, the tunable parameters include the latent dimension r and the three coefficients $\lambda_{1}$ , $\lambda_{2}$ , and $\lambda_{3}$ . We set r = [ τ min (m, n)], where τ∈[0,1] and [•] denotes the rounding function. Because there are many parameters, they may lead to overfitting. So, we set $\lambda_{2}$ and $\lambda_{3}$ to the same value to prevent overfitting. Finally, three parameters need to be determined, including τ , $\lambda_{1}$ , and $\lambda_{2}$ .

We choose to adopt a “fixing one and determining the others” strategy. First, we set τ to 0.1 and then picked the values of $\lambda_{1}$ and $\lambda_{2}$ from {0.001, 0.01, 0.1, 1} by LOOCV in a standard dataset. Then, we fix the determined values of $\lambda_{1}$ and $\lambda_{2}$ , and selected τ from {0.1,0.3,0.5,0.7,0.9,1}. The computational results for determining the $\lambda_{1}$ and $\lambda_{2}$ are listed in Table 1. We can discover that the AUC value reach maximum when $\lambda_{1}=0.1$ and $\lambda_{2}=0.01$ . As shown in Table 2, our model furnishes approximately the same good performance when τ≥0.7 . Therefore, we set τ=0.7 .

**TABLE 1:** The area under the curve (AUC) value using different $\lambda_{1}$ and $\lambda_{2}$ values in the leave-one-out cross validation (LOOCV).

*λ*_2_	0.001	0.01	0.1	1
*λ*_1_				
0.001	0.8667	0.8653	0.7689	0.6894
0.01	0.8849	0.8884	0.8798	0.7854
0.1	0.9067	0.9186	0.8968	0.8764
1	0.8932	0.8946	0.8937	0.8831

**TABLE 2:** The AUC value using different τ values while fixing $\lambda_{1}=0.1$ and $\lambda_{2}=0.01$ .

τ	0.1	0.3	0.5	0.7	0.9	1
AUC	0.8556	0.8721	0.8901	0.9186	0.9187	0.9186

The stopping criteria of the MSBMF algorithm are $f_{k}\leq tol_{1}$ and $\frac{\left|f_{k+1}-f_{k}\right|}{\max\left\{1,\left|f_{k}\right|\right\}}\leq tol_{2}$ , where $f_{k}=\frac{{\left\|S_{k+1}T_{k+1}-S_{k}T_{k}\right\|}_{F}}{{\left\|S_{k}T_{k}\right\|}_{F}}$ and $tol_{1}$ , $tol_{2}$ are the given tolerances. Here, according to the related studies (Yang et al., 2021), we set $tol_{1}=2\times 10^{-3}$ and $tol_{2}=10^{-4}$ .

### Comparison With Other State-of-the-Art Methods

**FIGURE 2:**
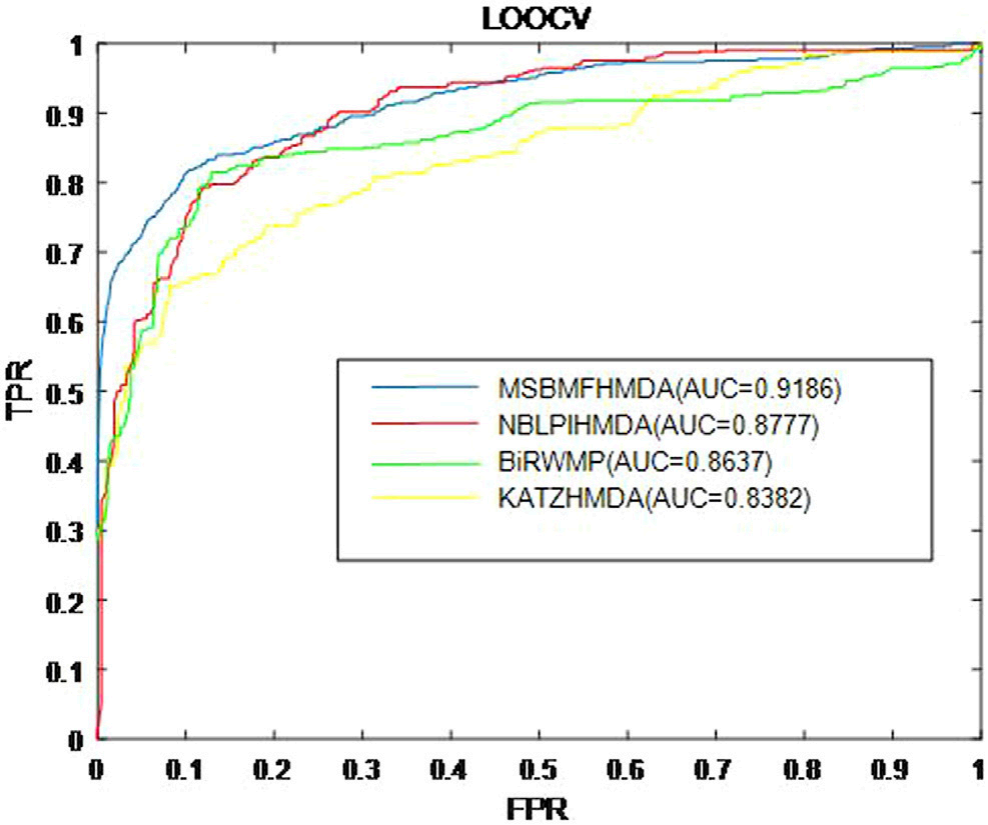
Prediction performance comparison between MSBMFHMDA and the other three methods in leave-one-out cross validation (LOOCV).

**FIGURE 3:**
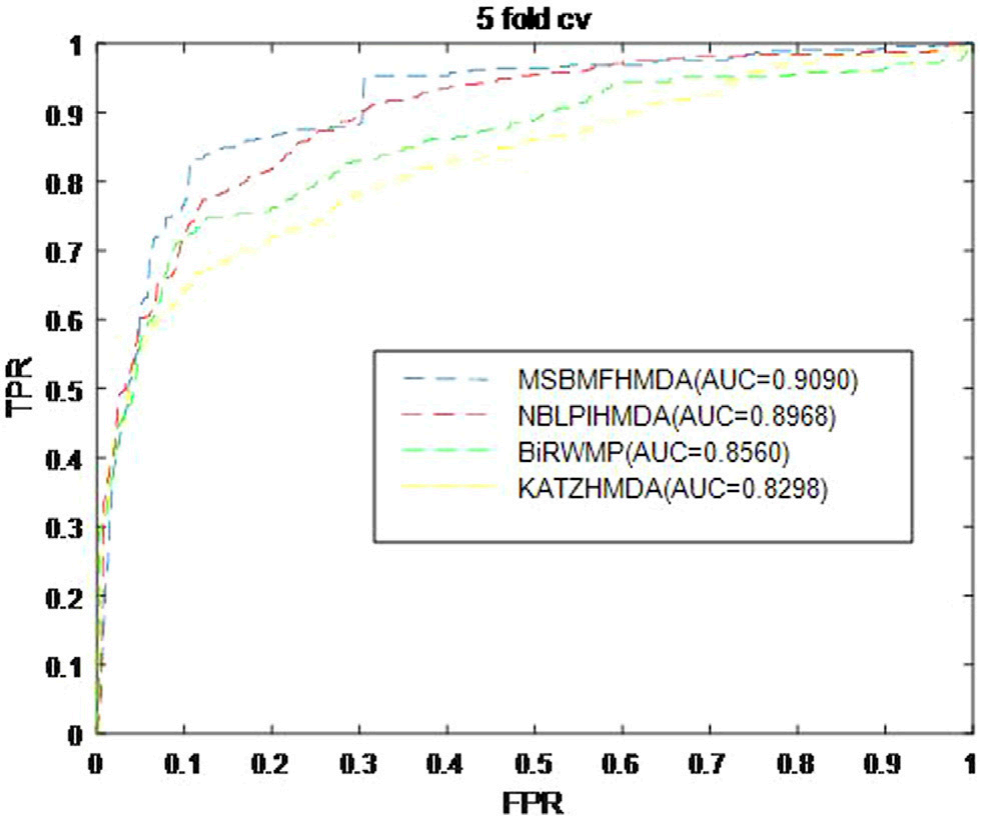
Prediction performance comparison between MSBMFHMDA and the other three methods in fivefold cross validation.

In this section, we consider several state-of-the-art microbe–disease association prediction methods and make comparisons to demonstrate superior performance of our proposed method MSBMFHMDA. We compare it with KATZHMDA, BiRWMP, and NBLPIHMDA based on the dataset of known microbe–disease associations. As illustrated in the following Figure 2 and Table 3, MSBMFHMDA yields best performance in LOOCV, achieving an AUC score of 0.9186, while KATZHMDA, BiRWMP, and NBLPIHMDA produce AUC scores of 0.8382, 0.8637, and 0.8777, respectively. As demonstrated in the following Figure 3, in the framework of fivefold cross validation, MSBMFHMDA can achieve a reliable AUC of 0.9043 ± 0.0048, which is better than the AUC achieved by KATZHMDA (0.8301 ± 0.0033), BiRWMP (0.8522 ± 0.0054), and NBLPIHMDA (0.8958 ± 0.0027).

**TABLE 3:** Performances of different methods in LOOCV and fivefold CV.

Method	LOOCV	Five-fold CV
MSBMFHMDA	0.9186	0.8993 ± 0.0032
NBLPIHMDA	0.8777	0.8958 ± 0.0027
BiRWMP	0.8637	0.8522 ± 0.0054
KATZHMDA	0.8382	0.8301 ± 0.0033

### The Sensitivity Analysis of Parameters

In this section, we concentrate on the sensitivity analysis for $\lambda_{1}$ , $\lambda_{2}$ , and τ in LOOCV. As we all know, when $\lambda_{1}=0.1,\lambda_{2}=0.01$ , and τ=0.7 , our model can realize excellent performance. We vary one parameter and keep the rest of the two parameters fixed to observe how the parameter benefits the AUC value.

**FIGURE 4:**
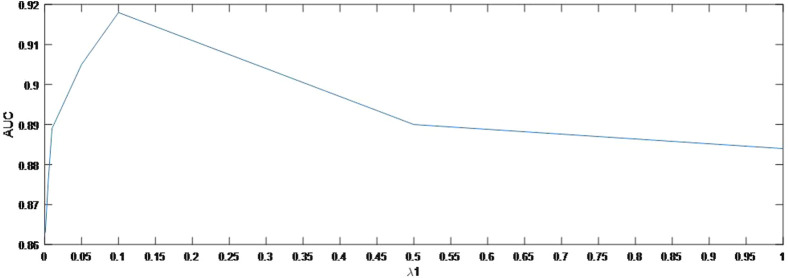
Variation of the AUCs with the various settings of $\lambda_{1}$ .

**FIGURE 5:**
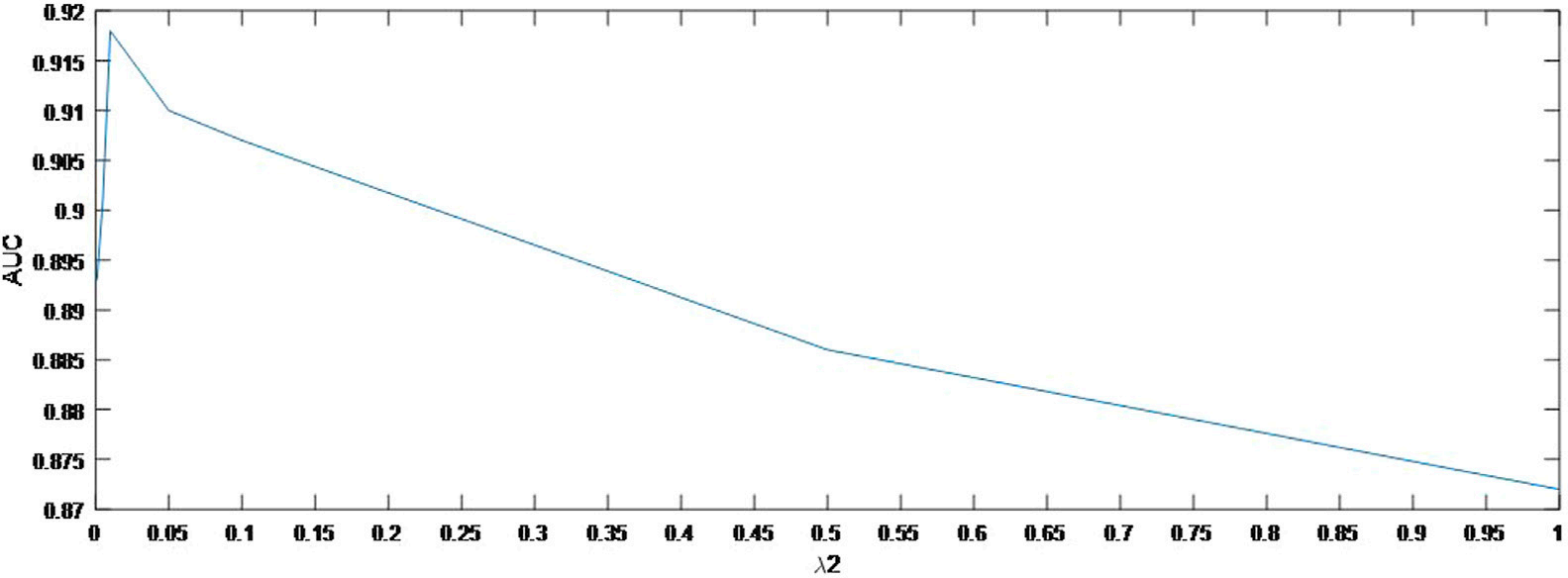
Variation of the AUCs with the various settings of $\lambda_{2}$ .

**FIGURE 6:**
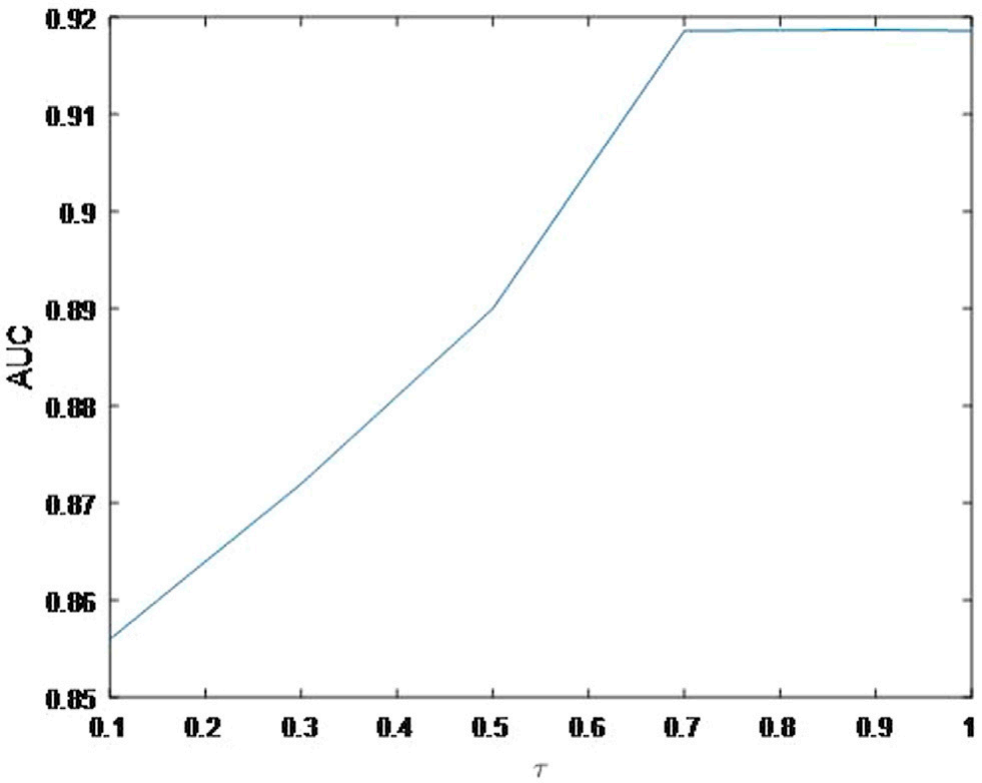
Variation of the AUCs with the various settings of τ .

As shown in Figure 4, the AUC can achieve the best values when $\lambda_{1}$ = 0.1. In the same way, Figure 5 indicates the best AUC on $\lambda_{2}$ = 0.01. Finally, the effect of parameter τ on the prediction accuracy is discussed. Figure 6 shows the AUC values of MSBMF with different τ . When τ > 0.7, the trend of AUC is becoming steady. If τ continue to increase to 0.9 or 1, our model will not only generate overfitting but also increases the computational complexity.

### Case Studies

Microbes are closely related to human health, and it is meaningful to explore whether microbes are associated with disease. In order to investigate into disease-causing microbes and further measure the prediction performance of our model, we selected three kinds of common microbe-induced diseases as cases for the analysis, namely, asthma, inflammatory bowel disease, and type 1 diabetes. The scores of the top 10 disease-related microbes are published in Supplementary Tables S1–S3, respectively.

Asthma is short for bronchial asthma, a heterogeneous disease characterized by chronic airway inflammation and airway hyper-responsiveness (Lemanske and Busse, 2010). The key features of asthma include chronic inflammation of the airway, high responsiveness of the airway to a variety of stimulators, limited variable reversible flow, and a series of changes with the course of the disease, namely, airway reconstruction (Çalışkan et al., 2013). Asthma is one of the most common chronic diseases in the world, with about 300 million people worldwide and about 45 million asthma patients in China, and there is a trend year by year. Epidemiological studies have shown that early exposure to microbes may determine the composition of the microbiome, which can help prevent allergies or cause the development of asthma. Asthma had been demonstrated to be closely associated with microbes by a number of research (Gilstrap and Kraft, 2013). In this section, though the there is implementation of our model to infer the novel asthma-related microbes, we published evidence for the top 10 potential asthma-related microbes predicted by MSBMFHMDA in Table 4.

**TABLE 4:** The validation results of the top 10 predicted asthma-related microbes by implementing MSBMFHMDA.

Rank	Microbe	Evidence
1	*Firmicutes*	PMID:23265859
2	*Clostridium difficile*	PMID:21872915
3	*Staphylococcus aureus*	PMID:17950502
4	*Bacteroides*	PMID:18822123
5	*Clostridium coccoides*	PMID:21477358
6	*Lachnospiraceae*	PMID:27433177
7	*Tropheryma whipplei*	PMID:26647445
8	*Lactobacillus*	PMID:20592920
9	*Actinobacteria*	PMID:23265859
10	*Enterobacteriaceae*	PMID:21639872

Inflammatory bowel disease (IBD) is a group of chronic non-specific intestinal inflammatory diseases that have no etiology, including ulcerative colitis and Crohn’s disease (D’Aoust et al., 2017). In this paper, we selected IBD as one of our case studies to evaluate the performance of our model. As illustrated in the following Table 5, there are 10 out of these top 10 microbes predicted by MSBMFHMDA that have been substantiated to be associated with IBD.

**TABLE 5:** The validation results of the top 10 predicted inflammatory bowel disease (IBD)-related microbes by implementing MSBMFHMDA.

Rank	Microbe	Evidence
1	*Clostridium coccoides*	PMID:21477358
2	*Prevotella*	PMID:24013298
3	*Lactobacillus*	PMID:20592920
4	*Bacteroidetes*	PMID:29492876
5	*Veillonella*	PMID:30573380
6	*Clostridium difficile*	PMID:21872915
7	*Firmicutes*	PMID:23265859
8	*Staphylococcus aureus*	PMID:17950502
9	*Helicobacter pylori*	PMID:22221289
10	*Actinobacteria*	PMID:23265859

Type 2 diabetes mellitus (T2D), also known as adult-onset diabetes, is characterized by a rise in blood sugar and a relative lack of insulin production because of a decline in the ability of insulin to help glucose enter cells for metabolism, a metabolic disorder resulting from a disorder of glucose metabolism (Furet et al., 2010). We took T2D as a case study for potential T2DM-related microbe prediction, and as illustrated in the following Table 6, 8 out of the top 10 predicted microbes were confirmed by experimental reports.

**TABLE 6:** The validation results of the top 10 predicted type 2 diabetes (T2D)-related microbes by implementing MSBMFHMDA.

Rank	Microbe	Evidence
1	*Clostridium difficile*	PMID:21872915
2	*Enterobacteriaceae*	PMID:21639872
3	*Staphylococcus aureus*	PMID:17950502
4	*Helicobacter pylori*	PMID:22221289
5	*Prevotella*	PMID:24013298
6	*Veillonella*	Unconfirmed
7	*Lachnospiraceae*	PMID:27433177
8	*Bacteroides*	PMID:18822123
9	*Burkholderia*	Unconfirmed
10	*Actinobacteria*	PMID:23265859

## Discussion and Conclusion

Since the application of traditional experimental methods to identify disease-associated microbes is time consuming and expensive, the calculation approach of MSBMFHMDA was put forward. Our model provides an effective scheme for dynamically integrating multiple similarities and extracting useful features to infer potential microbe–disease associations. The non-negative constraint in the model also ensures that the predicted scores of associations are non-negative. The computational results demonstrate that MSBMFHMDA has good performances for microbe–disease association prediction.

However, our model has two limitations. First, there are only 450 known microbe–disease associations, which accounts for a very small proportion of human microbe diseases. This may result in less comprehensive for prediction. Second, our method involves non-convex optimization, which leads to the local optimal solutions instead of the global optimal solution. In the future, we will reform predictive tasks based on the HMDAD record additional entries whether the quantity of microbial population is increased or decreased in the reported cases.

## Data Availability

The datasets presented in this study can be found in online repositories. The names of the repository/repositories and accession number(s) can be found in the article/Supplementary Material.
